# The Assessment of the Condition of Knee Joint Surfaces with Acoustic Emission Analysis

**DOI:** 10.3390/s21196495

**Published:** 2021-09-29

**Authors:** Bartłomiej Sawaryn, Natalia Piaseczna, Szymon Sieciński, Rafał Doniec, Konrad Duraj, Dariusz Komorowski, Ewaryst J. Tkacz

**Affiliations:** Department of Biosensors and Processing of Biomedical Signals, Faculty of Biomedical Engineering, Silesian University of Technology, Roosevelta 40, 41-800 Zabrze, Poland; natalia.piaseczna@polsl.pl (N.P.); szymon.siecinski@polsl.pl (S.S.); konrad.duraj@polsl.pl (K.D.); dariusz.komorowski@polsl.pl (D.K.); etkacz@polsl.pl (E.J.T.)

**Keywords:** knee examination, acoustic emission analysis, spectral analysis

## Abstract

The knee joint, being the largest joint in the human body, is responsible for a great percentage of leg movements. The diagnosis of the state of knee joints is usually based on X-ray scan, ultrasound imaging, computerized tomography (CT), magnetic resonance imaging (MRI), or arthroscopy. In this study, we aimed to create an inexpensive, portable device for recording the sound produced by the knee joint, and a dedicated application for its analysis. During the study, we examined fourteen volunteers of different ages, including those who had a knee injury. The device effectively enables the recording of the sounds produced by the knee joint, and the spectral analysis used in the application proved its reliability in evaluating the knee joint condition.

## 1. Introduction

Physical activity is one of the basic human activities performed every day. Maintained at the appropriate level for the individual, it ensures the preservation of a physical and psychological condition [1]. The human muscuoloskeletal system consists of a skeletal system and a muscular system [2]. Joints are the elements which connect the bones and may allow different degrees and types of movement [3].

The largest joint of the human skeleton is the knee joint, which connects the femur with the tibia and patella. It consists of the patella, ligaments, tendons, joint capsule, and joint cartilage. The joint cartilage has a thickness of 3–4 mm on average. The knee joint enables flexion and extension movements as well as rotational movement of the foot [3,4]. Due to the heavy loads, the knee joint is susceptible to injury [5,6].

The diagnosis of the state of knee joints is usually based on X-ray scan, ultrasound imaging, computerized tomography (CT), magnetic resonance imaging (MRI), or arthroscopy. The technological advancements in sensors encourage the development of simpler and cheaper devices for assessing the state of human joints. An example of such an approach is Acoustic Emission Analysis (AEA) [5,6,7,8].

Acoustic Emission Analysis is a widely used tool in evaluating the condition ofjoints [5,7,9,10,11,12]. Schueter at al. identified potential osteoarthritis (OA) biomarkers based on the selection of promising candidates for high frequency acoustic emission (AE) measurements generated during knee movement with load. The study shows how measuring AE during simple sit-stand-sit movements can be used to generate new OA biomarkers. AE measurements likely reflect a combination of structural changes and joint stress factors [13].

The aim of the study was to construct an inexpensive, portable device for assessing the condition of human knee joints. The choice of the knee joint is justified by the fact that it is one of the largest human joints and the cartilage surface is close to the skin.

## 2. Materials and Methods

### 2.1. Study Group

The study group consisted of 14 subjects described as follows:

- Subject 1: Gender: Male, age: 89, height: 174 cm, body mass: 61 kg, lifestyle: Active, physical condition: High, knee injuries: No, other remarks: Dog walking, gardening;
- Subject 2: Gender: Female, age: 81, height: 165 cm, body mass: 67 kg, lifestyle: Active, physical condition: Average, knee injuries: No, other remarks: Gardening, nordic walking;
- Subject 3: Gender: Male, age: 23, height: 187 cm, body mass: 95 kg, lifestyle: Active, physical condition: Average, knee injuries: Rupture of joint capsule and patella, other remarks: Skating and running;
- Subject 4: Gender: Male, age: 36, height: 180 cm, body mass: 92 kg, lifestyle: Sedentary-standing, physical condition: Average, knee injuries: Multiple contusions of the knee related to off-road riding on a motorcycle, other remarks: Irregular physical activity;
- Subject 5: Gender: Male, age: 23, height: 187 cm, body mass: 70 kg, lifestyle: Standing, physical condition: Average, knee injuries: No, other remarks: Gym;
- Subject 6: Gender: Male, age: 37, height: 189 cm, body mass: 88 kg, lifestyle: Sedentary-standing, physical condition: Average, knee injuries: Rupture of cruciform ligaments in the right knee, other remarks: Cycling, gym;
- Subject 7: Gender: Male, age: 24, height: 183 cm, body mass: 83 kg, lifestyle: Active, physical condition: High, knee injuries: No, other remarks: Swimming and skiing;
- Subject 8: Gender: Female, age 54, height: 164 cm, body mass: 90 kg, lifestyle: Sedentary-standing, physical condition: Average, knee injuries: Right knee injury related to skiing, other remarks: Swimming, irregularly; spinal disc herniation at L2–L3 level;
- Subject 9: Gender: Male, age: 51, height: 184 cm, body mass: 102 kg, lifestyle: Active, physical condition: High, knee injuries: Right knee arthroscopy, ACL and medial meniscus resection, other remarks: Strength sports and alpine skiing;
- Subject 10: Gender: Female, age: 83, height: 158 cm, body mass: 62 kg, lifestyle: Active, physical condition: Average, knee injuries: No, other remarks: Gymnastics and aqua-aerobics;
- Subject 11: Gender: Female, age: 55, height: 160 cm, body mass: 59 kg, lifestyle: Active, physical condition: High, knee injuries: No, other remarks: Gymnastics and cycling;
- Subject 12: Gender: Female, age: 25, height: 164 cm, body mass: 54 kg, lifestyle: Active, physical condition: High, knee injuries: Knee injury (unspecified), other remarks: Running and strength sports;
- Subject 13: Gender: Female, age: 23, height: 162 cm, body mass: 67 kg, lifestyle: Sedentary-standing, physical condition: Average, knee injuries: No, other remarks: Gym since six months;
- Subject 14: Gender: Male, age: 42, height: 189 cm, body mass: 100 kg, lifestyle: Active, physical condition: Average, knee injuries: Numerous injuries and surgeries of the knee joints, other remarks: Cycling and motorbike riding.

Each subject included in the study group filled in a questionnaire with questions on the gender, age, height, body mass, lifestyle (sedentary, standing, sedentary-standing, active), physical activity (kind of activity, time, level), physical condition, musculoskeletal system injuries, and additional remarks.

### 2.2. Measuring Devices

For this study, we designed a device for measuring the acoustic emissions in knee joints which consists of:

- two USB sound cards;
- battery basket;
- two microphones:
- jack connectors;
- cables.

Due to the wide application, availability and price, an electret microphone with the following parameters was chosen for this project [14]:

- Supply voltage: 3.3 V;
- 20 dB amplification (10×);
- Temperature range: −40 °C÷85 °C;
- Dimensions: 15 mm × 9 mm;
- Built-in noise filter;
- Voltage stabilizer: 3.3 V: XC6206 (662K).

**Figure 1:**
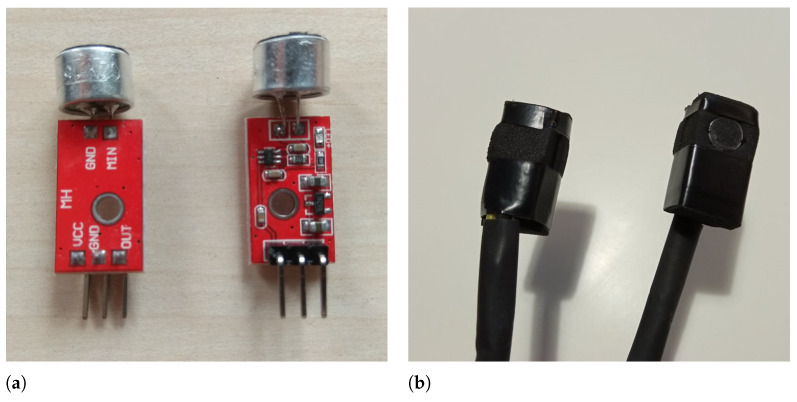
The microphone used in the project. (**a**) without protective coat; (**b**) with protective coat.

The microphone covers the range of audible sounds (16–16,000 Hz). An additional advantage was the easy adaptation of the position of the microphone head in such a way that it adheres to the skin surface as much as possible. Additionally each microphone was isolated from the environment by coating it with a hard sponge. The sponge was placed in a way that did not disturb signal acquisition. The microphone used in this work is presented in the Figure 1.

**Figure 2:**
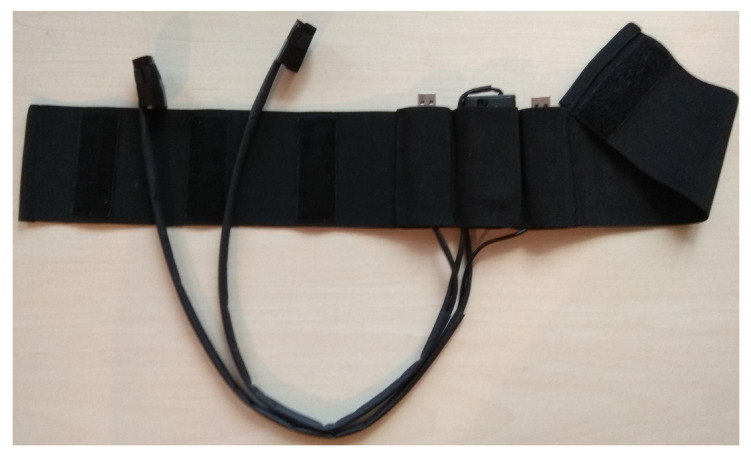
Constructed device.

Figure 2 shows the constructed device. Sound cards and battery basket are inserted into pockets in the elastic band.

The elastic band was used for ensuring the exact placement of microphones on the skin. The device is designed and constructed so that it fits most people and it does not require calibration.

### 2.3. Experiment

The experiment was approved by the Bioethics Committee of the Medical University of Silesia under the resolution number KNW/0022/KB1/79/18 taken on 16 October 2018. All the participants gave the informed consent before the experiment.

**Figure 3:**
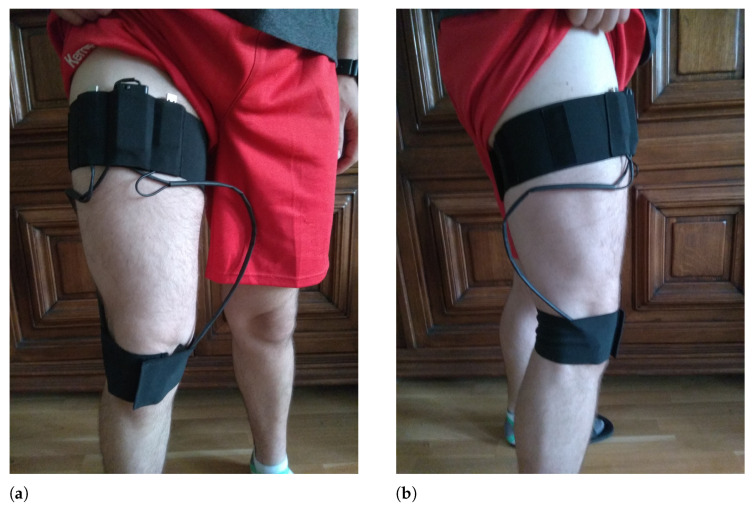
Device placement. (**a**) frontal view; (**b**) sagittal view.

The test consisted of ten cycles of full knee flexion and extension at moderate speed in a sitting position while wearing the recording device. The microphones were placed on both sides of the knee (see Figure 3).

**Figure 4:**
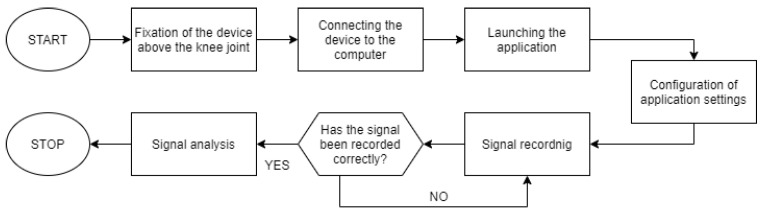
Block diagram describing the operation of the application.

Based on the collected information about the course of the knee examination, we developed the procedure algorithm shown in Figure 4.

The device is not intended for independent use. Another person is required to operate the device and the application. To properly examine the knee joint, the following steps should be performed in the following order:

1. Fix the device above the knee joint and connect to the computer.
2. Start up the application and configure the settings.
3. Ask the subject to perform a series of movement (in case of our study—ten cycles of full knee flexion and extension at moderate speed in a sitting position).
4. Check the signal. If the signal is recorded properly—go to the next step. Otherwise, repeat the signal acquisition.

It is essential that the examination is carried out in a noise-free environment. During the examination it is necessary to avoid talking, moving furniture, walking, etc. We recommend to use the device in a room with a noise level up to 35 dB, which is equivalent to a whisper. Any object that could potentially restrict the patient movements should also be removed from the surroundings.

After properly recording the signal, the application proceeds to the analysis.

### 2.4. Signal Processing

Acoustic waves generated by human joints fluctuate over time [15]. Thus, the analysis of such signals should consider more factors than merely measuring the length, period, or amplitude—which provides insufficient data to extract useful information from the signal [16]. For evaluating the data obtained in our study, we chose to use spectral analysis [17,18]. It consists in converting the signal to an image in the frequency domain. Such an operation enables the observation of frequency components which appear in the signal.

There are several methods widely used for signal spectral analysis [19]. For this work, we chose to use the following:

1. Fast Fourier Transform (FFT)—this method helps to examine of what frequencies the original function consists. FFT uses the basic periodic sine and cosine functions. Calculations are fast due to the breakdown of the algorithm into shorter and simpler operations [20];
2. Spectrogram—by this operation we create a graphical representation of the frequencies present in time-varying signal [21]. For this study, the signal must be previously filtered (we used a Butterworth bandpass filter in the range 0.15–10 kHz). To analyze the results, it is also worth comparing the spectrogram for low (0.15–3.5 kHz), medium (3.5–7.5 kHz) and high (7.5–10 kHz) frequencies to evaluate changes in each range frequency (filter bank can be used for this operation);
3. Periodogram is used to estimate the spectral power density using the discrete Fourier transform [22];
4. Welch Spectrum—a method used to approximate the power of a signal spectrum at different frequencies [17]. It is an improved periodogram method enriched with the Bartlett method. As a result, the noise in the spectrum is significantly reduced, but at the expense of lower frequency resolution;
5. AR Spectrum—an autoregressive parametric method, which uses data as the result of a linear operation [23]. On their basis, the parameters of the created linear model are estimated;
6. Wavelet transform—a scalar product of the signal with the selected base function [24]. Thanks to the possibility of scaling and shifting the base function, it allows to analyze the change of the signal frequency over time, which is not possible in the case of the Fourier transform. It is a supplementary method rather than the main analytical function.

The calculated spectra have to be converted into a decibel scale which enables easier analysis and comparison. Moreover, based on the obtained spectra, their total and partial powers should be calculated, corresponding to the frequencies given in the description of the spectrogram. Then, to create coefficients that enable the assessment of the contribution of bandwidth to the overall signal strength, the spectral power in the partial bands must be compared to the entire signal band.

## 3. Evaluation of the Operation of the Device

In order to verify the correctness of the work of the designed device and the dedicated application, it was tested according to the scenario developed for this circumstance. It consists of the following:

1. Verification of the work of the constructed device;
2. Verification of the work of the application recording the acoustic signal;
3. Verification of the work of the application analyzing the acquired signal.

The correctness of the designed software was validated on a computer equipped with the following configuration:

- CPU: Intel Core i7-6500U, 2.5 G–2.6 GHz
- 16 GB RAM,
- Hard drive: 460 GB SSD,
- Operating system: Microsoft Windows 10 Home (64-bit),
- MATLAB R2014a environment.

### 3.1. Device Tests

To verify the work of constructed device, all connections have to be checked with a voltmeter and the device needs to be connected to the computer. Then, the visibility of external sound cards to the operating system should be verified.

Next, we recorded the sound signals from the surface of the knee using both microphones and a built-in sound recorder. The signals were played out—variable sounds from the area of the knee joint were coming out of the speakers.

### 3.2. Verification of the Work of the Application Recording the Acoustic Signal

**Figure 5:**
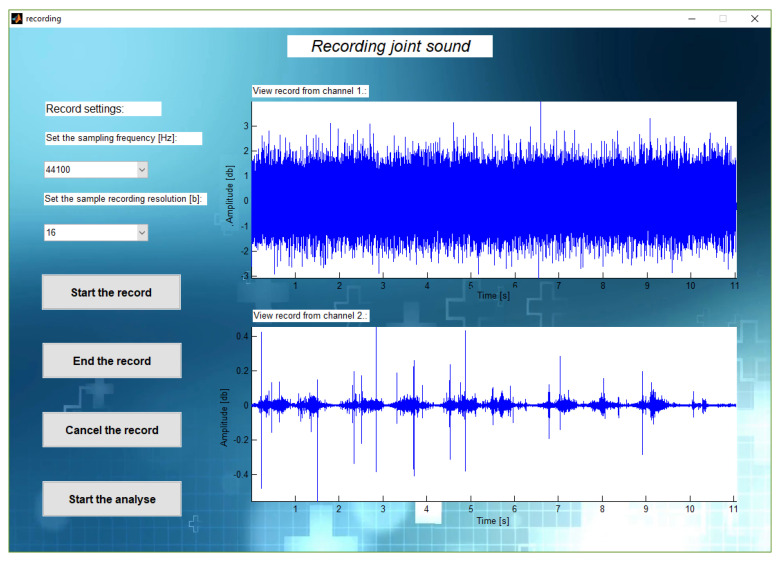
An example of a correctly received signal and an error while connecting the device.

For evaluating the work of the developed application we recorded two signals and displayed them in the app window. Figure 5 shows the screenshot of the window with recorded signals—in the first channel, the Jack connector is disconnected from the sound card and in the second channel the sound is obtained correctly. The visual evaluation of the signal was sufficient to establish that the software works properly.

### 3.3. Verification of the Work of the Application Analyzing the Acquired Signal

**Figure 6:**
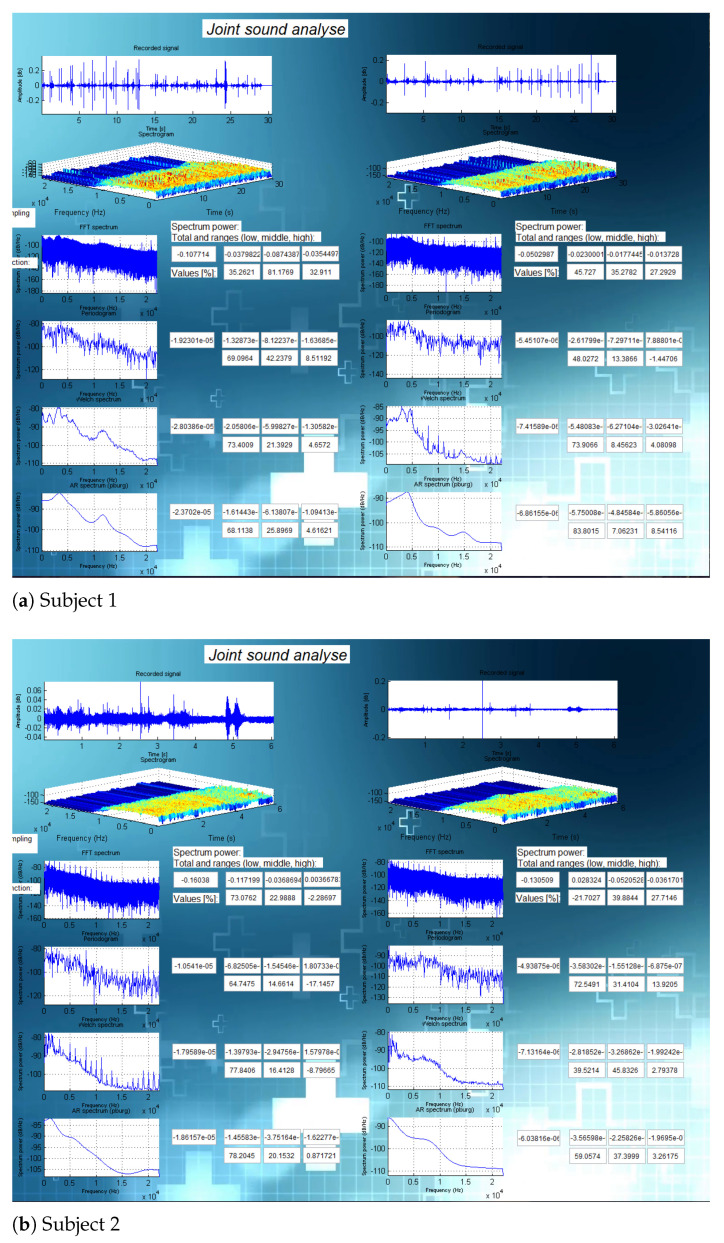
Test study analysis comparison.

Verification of this part consisted of two stages. First, several attempts were made to analyze signals taken from different people to determine if the application shows different results for different subjects. Two sample results are presented in Figure 6.

**Figure 7:**
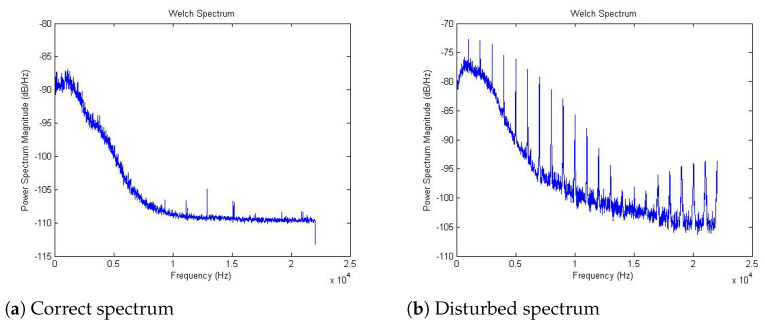
Comparison of signal spectra.

At the present stage the signals recorded by the device may be disturbed. The interference is visible in the calculated signal spectra. Figure 7 shows repeated frequency components present in the disturbed signal in comparison with properly recorded one.

This situation may be caused by the imperfect design of the device, but also by the artifacts transferred from another device near the test apparatus during the recording. The artifacts do not affect the visual evaluation of the spectrum, and the frequency peaks that appear in the graph did not affect significantly the spectrum power calculation.

After a successful part of the tests, the same subjects were recorded, but when bending the knee joint, a pen was opened and closed near one of the microphones. We proposed simulating the sounds of malfunctioning knee joints with the sound from a ballpoint pen because of the similar intensity and frequency components to the sounds generated by knee joints. Figure 7 shows the comparison between the correct signal and its spectrum, and a disturbed signal along with its spectrum. Both measurements were made on the same person.

A significantly higher amplitude of the simulated disturbed signal is noticeable and the correct signal spectrum is much smoother and has a different shape.

Table 1 presents the summary of the calculated spectral powers for normal and abnormal signals in the total, low, medium, and average frequency band. The values confirm the differences between this two signals.

## 4. Results

**Figure 8:**
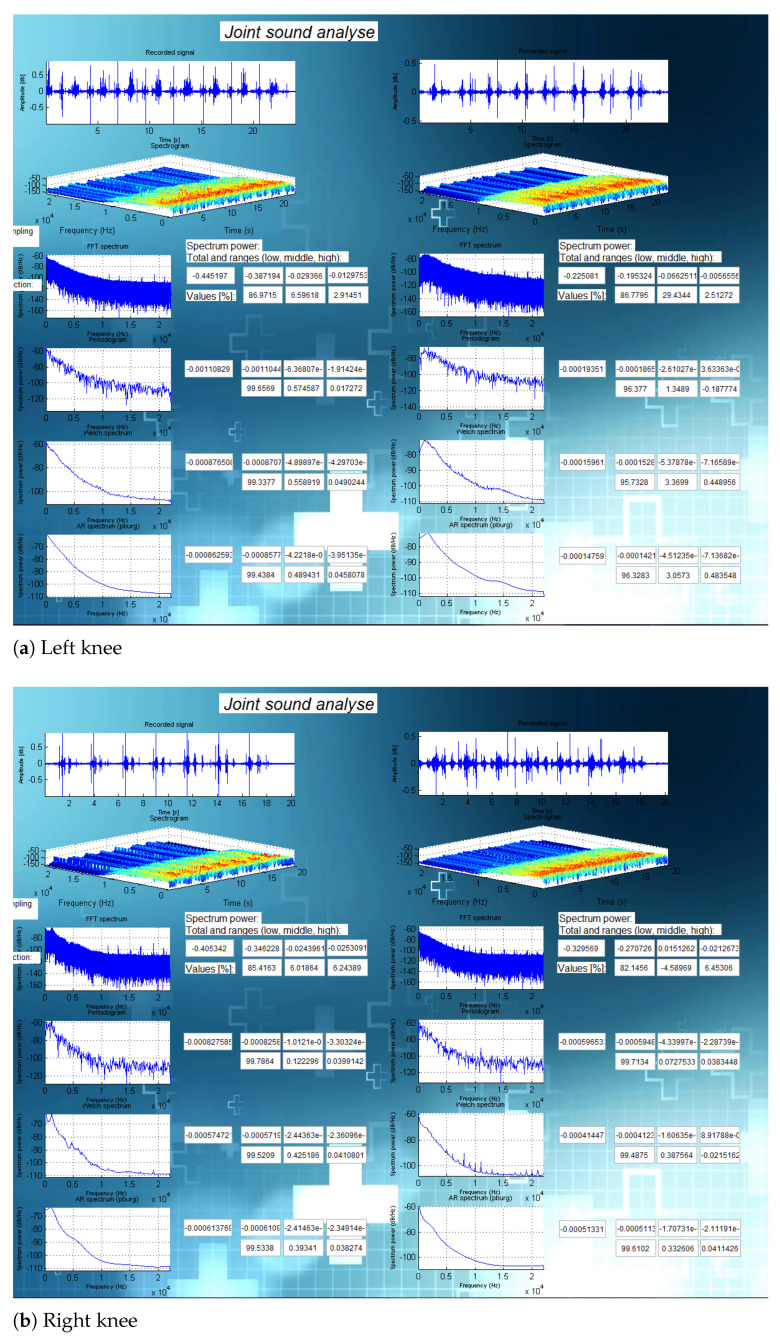
Analysis for a young and healthy person (subject 6).

**Figure 9:**
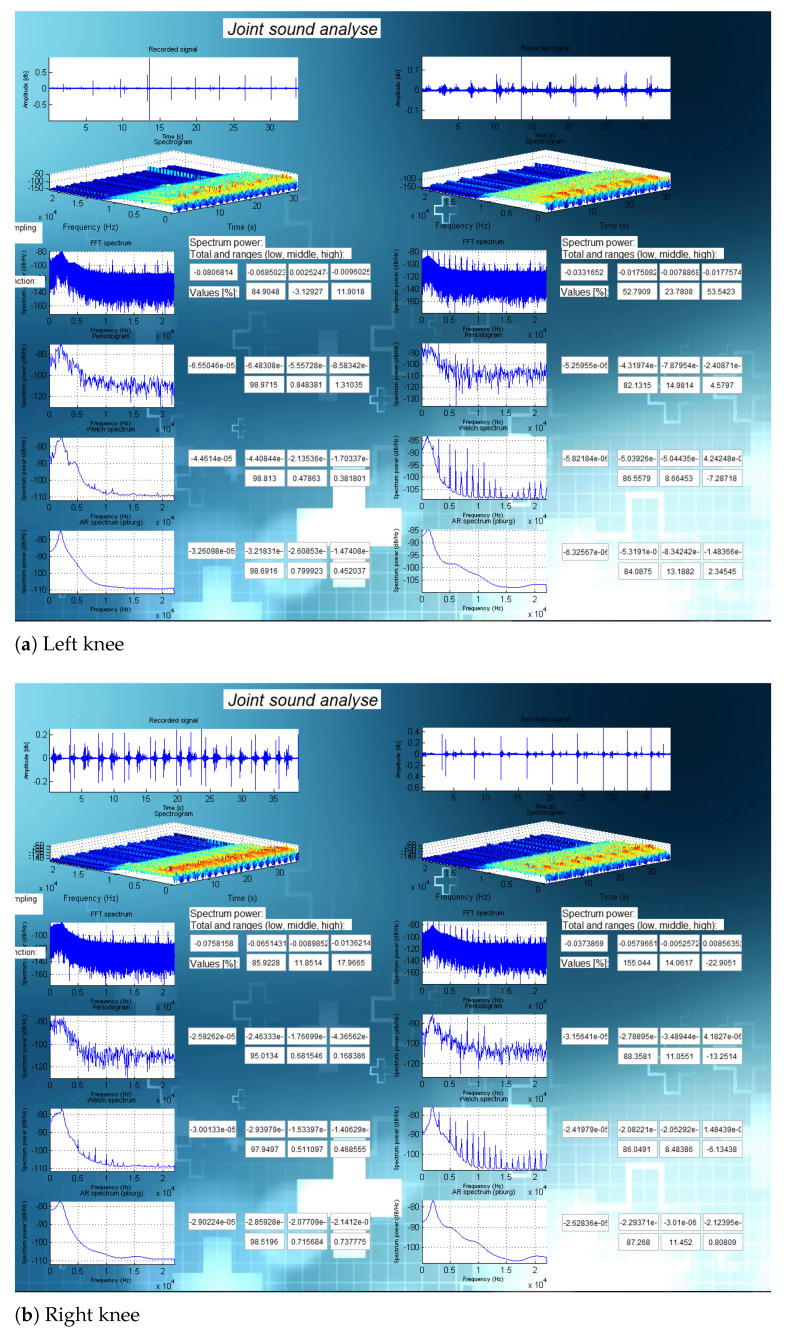
Analysis for an older person (subject 2).

**Figure 10:**
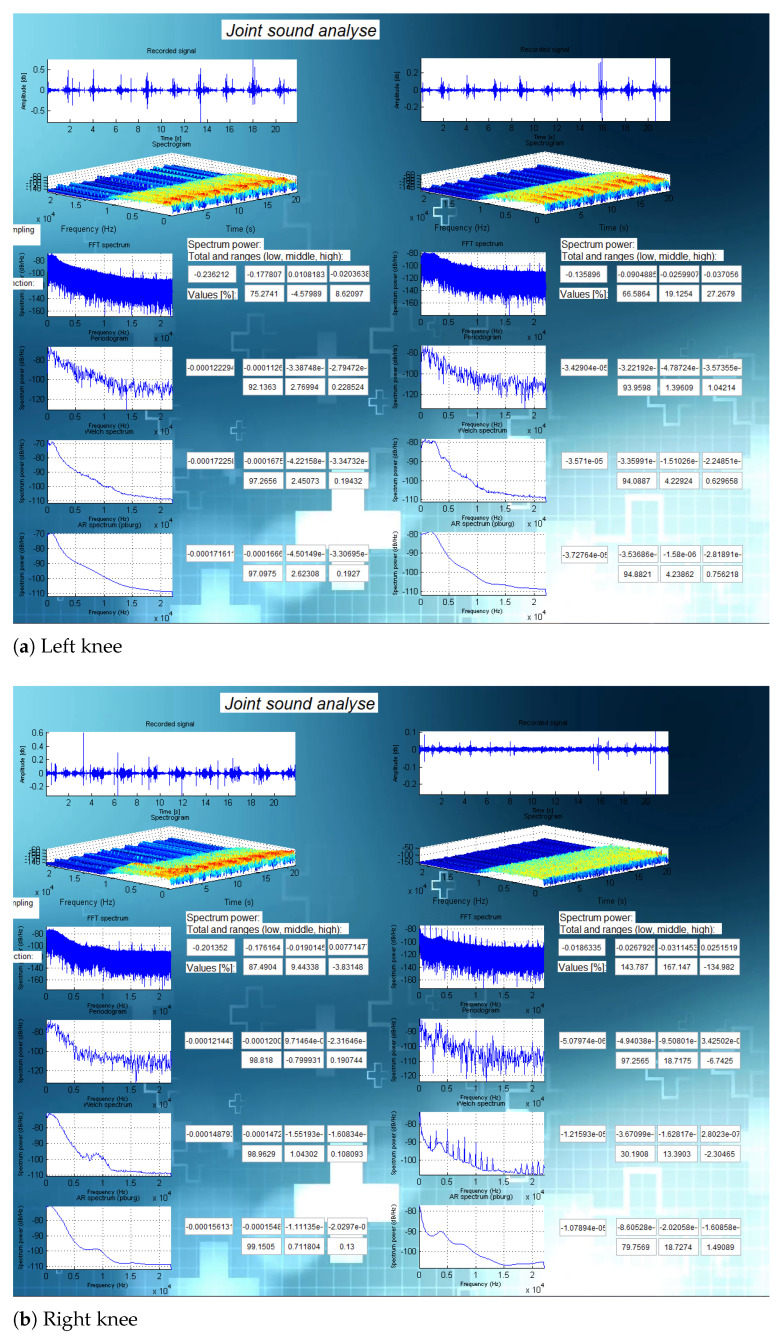
Analysis for a person after multiple knee injuries and surgeries (subject 14).

In this section, we present the results of the analysis for three specific cases. Figure 8 presents the results of an analysis on subject 6 (24-year old healthy male subject with no knee injury, maintaining an active lifestyle), Figure 9 presents the results of an analysis on subject 2 (healthy and active 81-year-old female subject), and Figure 10 shows the analysis on subject 14 (42-year old male subject after multiple knee injuries and surgical procedures).

The analyses for all subjects (including those shown in Figure 8, Figure 9 and Figure 10 are presented in Appendix A.

**Figure 11:**
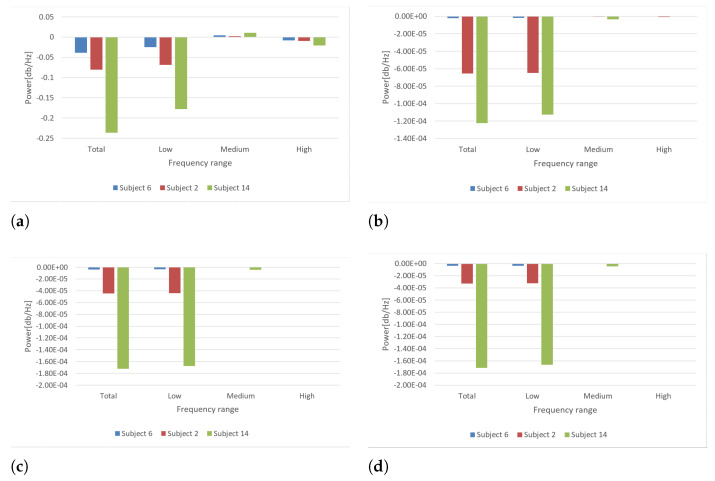
Power graphs of signal spectra for three chosen subjects. (**a**) FFT; (**b**) Periodogram; (**c**) Welch; (**d**) AR.

Table 2 and Figure 11 present the comparison of the calculated spectral powers for three different subjects measured by the left microphone on the left knee. Spectral powers were calculated in four different frequency bands: Total, low, medium, and high.

There are visible differences between the calculated values for a young and healthy person, a healthy older person, and the person after multiple knee injuries.

## 5. Discussion

We created an inexpensive and portable device that allows to record the two-channel acoustic signal produced by the knee joint during leg movements. We also developed a dedicated application for acquiring, saving, and analyzing the signal. The apparatus can be used for joint examinations with and without the load. The small dimensions of the device and its mobility allow for a wide diagnostic application. An additional advantage of the device is the fact that it is comfortable, easy to put on, and the examination can be performed by the patient at home. Moreover, the device can be an alternative to expensive imaging techniques or palpation examination.

The acoustic signal from the knee joint is susceptible to interference. To reduce the interference, the elements of the device (elastic bands and microphones) should be covered with more adhesive material, e.g., silicone strips. Such an alteration would prevent the device from rubbing on the skin and generating additional noise. It is important to remain relatively quiet during the test (avoid talking, moving pieces of furniture, etc.). The device is equipped with a protective foam coat that suppresses some of the interference from the environment. However, we have not tested the device in noisy rooms at this moment.

The recorded signal is unique for each examined subject. Additionally, despite the similarities in signal and spectrum, the information collected and processed from the knee joints of one person from two limbs is different.

The most prominent changes are caused by the age and musculoskeletal system injuries. The spectrum calculated for the elderly differs from standard spectra, especially in the mid-frequency range, and for people who suffered from knee injuries—in the high frequency range. This is also confirmed by a study performed by Song et al. [25].

The most significant influence on the acoustic signal is caused by the work of joint ligaments. The influence of the friction of the articular surfaces is less significant, but its impact is still noticeable.

The device, together with the developed application, has high potential to support the work of orthopedists and compare different groups of patients (after injuries, with joint abnormalities, after joint reconstruction, for athletes, etc.) among themselves. Based on this study, the device may find its use for the screening the condition of the knee joint and to indicate the need of a more specialized and personalized treatment.

Our device and examination technique differs from reported earlier; Whittingslow et al. evaluated knee joints by using only one sound sensor and took into account only patient’s age as a factor influencing joint condition [26]. In our work we consider more factors, such as injuries and lifestyle.

Feng et al. used advanced sensor (piezoelectric film) for monitoring joint condition [12]. This type of sensor may increase the costs of the device, whilst our device uses inexpensive and easily available microphone.

Further work should concentrate on widening the knowledge and signal pool in order to increase the diagnostic capabilities of the device in different groups of patients with different medical conditions. We also consider increasing the number of sensor in order to create a microphone array that will enable mapping of the joint. The proposed method may become a valuable diagnostic guide to supplement the medical diagnosis at an early stage of care.

## 6. Conclusions

The aim of the project was to develop a device to analyze the acoustic signal that would allow the assessment of the condition of human articular surfaces. The device was designed with the selected electronic components and the software was implemented in MATLAB R2014a. The designed application comes with a user interface that allows to configure the settings for recording the signal as well as its analysis. The developed application is relatively simple, easy to read, and intuitive to use. After the first configuration, the software no longer requires user participation until the analysis process is complete.

Tests carried out both during the development of the program and after its completion, allowed to quickly get rid of its defects, anticipate possible exceptions, and secure the app against their occurrence. The verification steps confirmed the correct operation of the application.

The performed analysis shows differences between spectral power for a young and healthy subject, an elderly and active subject, and a subject after several knee injuries and surgeries. The analysis allowed to clearly distinguish these specific cases without any calibration.

The possibility of further expanding the project with additional functionalities and improvements was noticed, which were not known at the stage of development of the work objective. This could be, for example, the use of hydrophones or more accurate sound cards.

In future studies, we will consider further development of the device and software, such as adding the reference in the form of imaging (ultrasonography, CT, MRI), increasing the number of subjects during the experiments, consultations with healthcare facilities specializing in orthopedics, and performing similar analyses for other human joints.

## Figures and Tables

**Table 1:** Spectral powers for normal and abnormal signals.

Spectrum	Scope	Normal	Abnormal
FFT	Total	–0.0384558	–0.19035
	Low	–0.0250778	–0.0886131
	Medium	0.00421264	–0.0683395
	High	–0.008172	–0.0847173
Periodogram	Total	$-2.28\times 10^{-6}$	$-5.27\times 10^{-5}$
	Low	$-1.99\times 10^{-6}$	$-3.40\times 10^{-5}$
	Medium	$-3.68\times 10^{-7}$	$9.12\times 10^{-6}$
	High	$-3.05\times 10^{-7}$	$-2.18\times 10^{-6}$
Welch	Total	$-3.99\times 10^{-6}$	$-5.06\times 10^{-5}$
	Low	$3.73\times 10^{-6}$	$-4.08\times 10^{-5}$
	Medium	$-1.74\times 10^{-7}$	$-4.14\times 10^{-6}$
	High	$-1.31\times 10^{-7}$	$-7.78\times 10^{-6}$
AR	Total	$-3.71\times 10^{-6}$	$-5.42\times 10^{-5}$
	Low	$-3.45\times 10^{-6}$	$-5.42\times 10^{-5}$
	Medium	$-1.10\times 10^{-7}$	$-9.65\times 10^{-6}$
	High	$-1.43\times 10^{-7}$	$-5.71\times 10^{-6}$

**Table 2:** Spectral powers for three different subjects (left knee, microphone on the left).

Spectrum	Scope	Subject 6	Subject 2	Subject 14
FFT	Total	$-3.85\times 10^{-2}$	$-8.07\times 10^{-2}$	$-2.36\times 10^{-1}$
	Low	$-2.51\times 10^{-2}$	$6.85\times 10^{-2}$	$-1.78\times 10^{-1}$
	Medium	$4.21\times 10^{-3}$	$2.52\times 10^{-3}$	$1.08\times 10^{-2}$
	High	$-8.17\times 10^{-3}$	$-9.60\times 10^{-3}$	$-2.04\times 10^{-2}$
Periodogram	Total	$-2.28\times 10^{-6}$	$-6.55\times 10^{-5}$	$-1.22\times 10^{-4}$
	Low	$-1.99\times 10^{-6}$	$-6.48\times 10^{-5}$	$-1.13\times 10^{-4}$
	Medium	$-3.68\times 10^{-7}$	$-5.56\times 10^{-7}$	$-3.39\times 10^{-6}$
	High	$-3.05\times 10^{-7}$	$-8.58\times 10^{-7}$	$-2.79\times 10^{-7}$
Welch	Total	$-3.99\times 10^{-6}$	$-4.46\times 10^{-5}$	$-1.72\times 10^{-4}$
	Low	$-3.73\times 10^{-6}$	$-4.41\times 10^{-5}$	$-1.68\times 10^{-4}$
	Medium	$-1.74\times 10^{-7}$	$-2.14\times 10^{-7}$	$-4.22\times 10^{-6}$
	High	$-1.31\times 10^{-7}$	$-1.70\times 10^{-7}$	$-3.35\times 10^{-7}$
AR	Total	$-3.71\times 10^{-6}$	$-3.26\times 10^{-5}$	$-1.72\times 10^{-4}$
	Low	$-3.45\times 10^{-6}$	$-3.22\times 10^{-5}$	$-1.67\times 10^{-4}$
	Medium	$-1.10\times 10^{-7}$	$-2.61\times 10^{-7}$	$-4.50\times 10^{-6}$
	High	$-1.43\times 10^{-7}$	$-1.47\times 10^{-7}$	$-3.31\times 10^{-7}$

**Figure A1:**
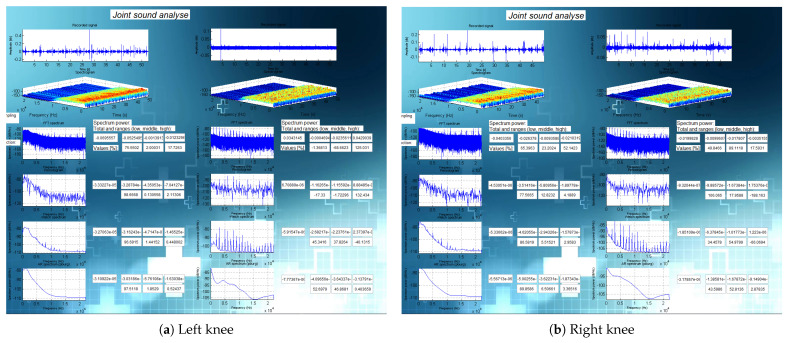
Analysis for subject 1.

**Figure A2:**
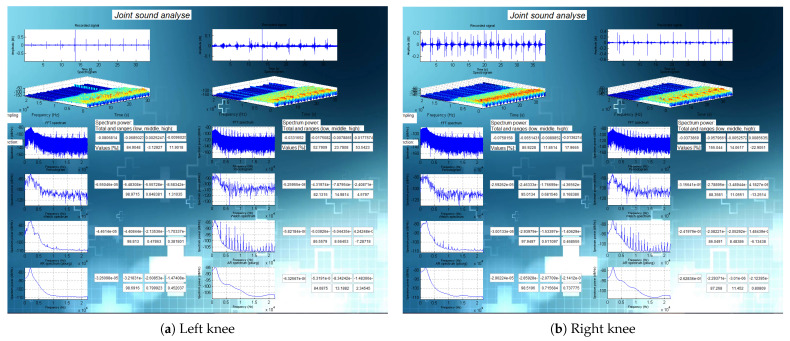
Analysis for subject 2.

**Figure A3:**
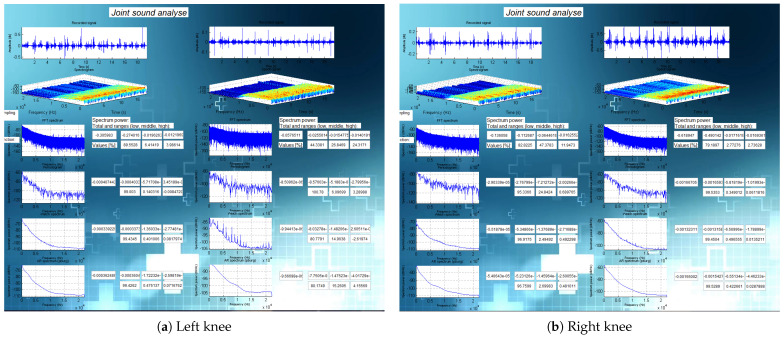
Analysis for subject 3.

**Figure A4:**
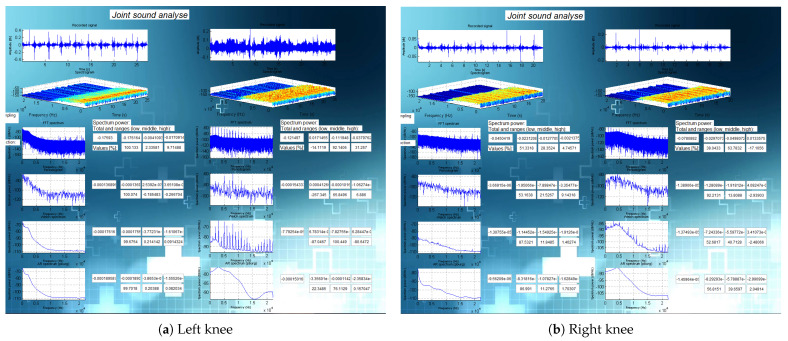
Analysis for subject 4.

**Figure A5:**
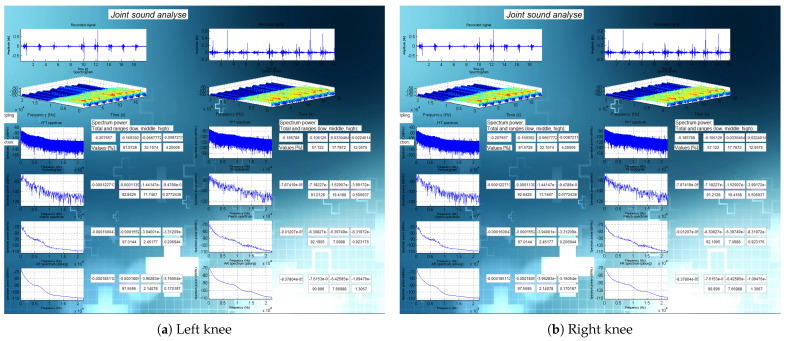
Analysis for subject 5.

**Figure A6:**
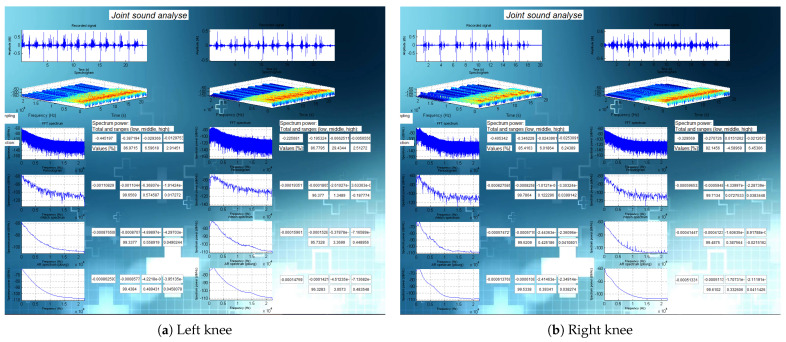
Analysis for subject 6.

**Figure A7:**
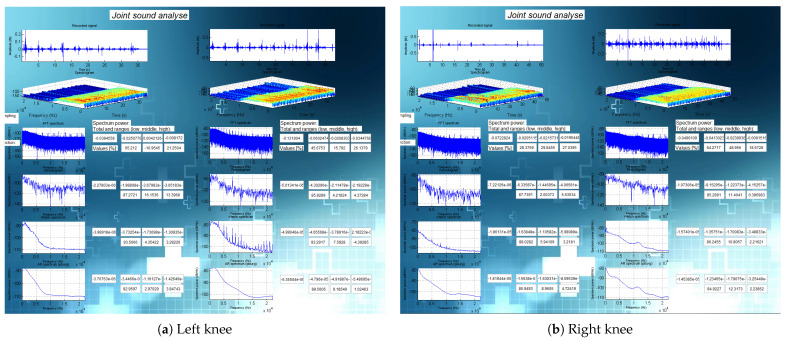
Analysis for subject 7.

**Figure A8:**
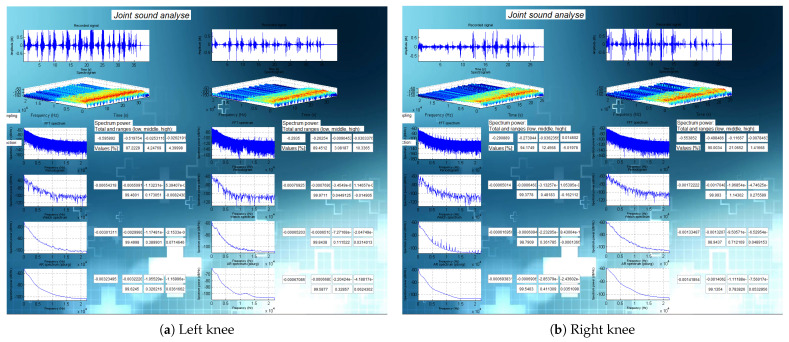
Analysis for subject 8.

**Figure A9:**
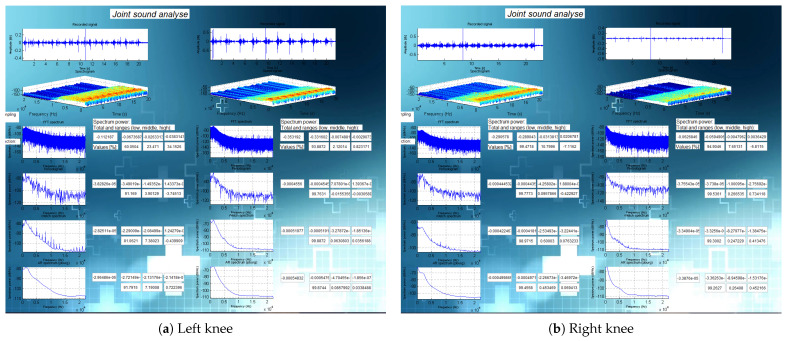
Analysis for subject 9.

**Figure A10:**
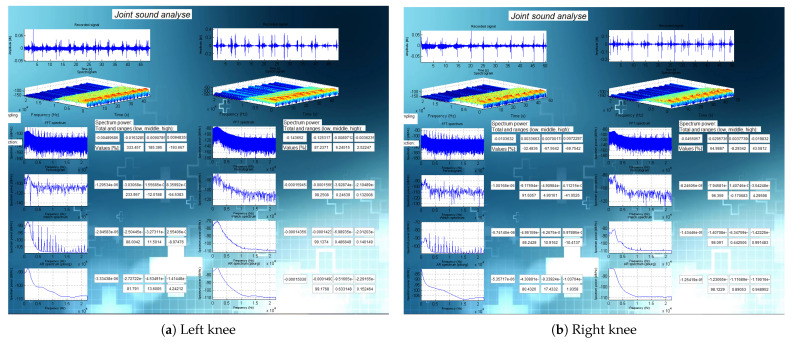
Analysis for subject 10.

**Figure A11:**
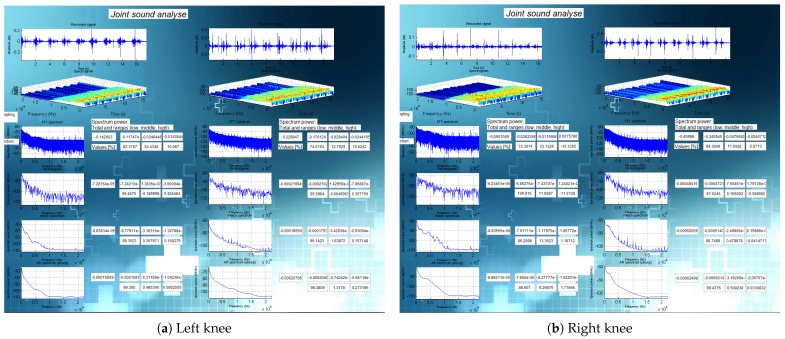
Analysis for subject 11.

**Figure A12:**
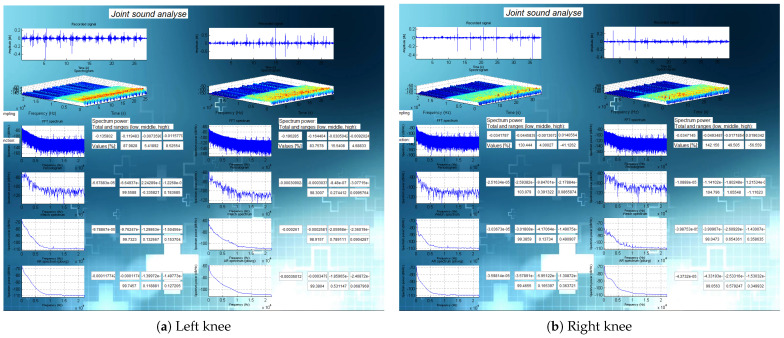
Analysis for subject 12.

**Figure A13:**
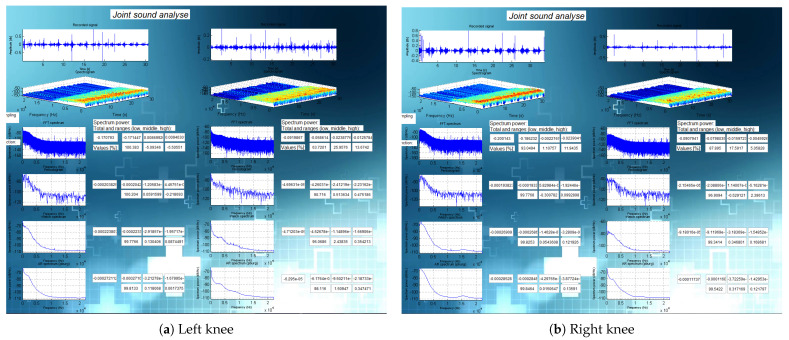
Analysis for subject 13.

**Figure A14:**
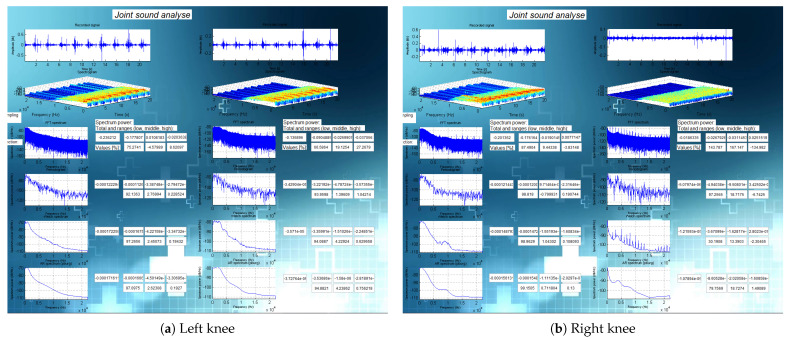
Analysis for subject 14.

## Data Availability

Research data are available on request from the authors.

## Appendix A. Analysis for All 14 Subjects

1. gender: Male, age: 89, height: 174 cm, body mass: 61 kg, lifestyle: Active, physical condition: High, knee injuries: No, other remarks: Dog walking, gardening (Figure A1);

2. gender: Female, age: 81, height: 165 cm, body mass: 67 kg, lifestyle: Active, physical condition: Average, knee injuries: No, other remarks: Gardening, nordic walking (Figure A2);

3. gender: Male, age: 23, height: 187 cm, body mass: 95 kg, lifestyle: Active, physical condition: Average, knee injuries: Rupture of joint capsule and patella, other remarks: Skating and running (Figure A3);

4. gender: Male, age: 36, height: 180 cm, body mass: 92 kg, lifestyle: Sedentary-standing, physical condition: Average, knee injuries: Multiple contusions of the knee related to off-road riding on a motorcycle, other remarks: Irregular physical activity (Figure A4);

5. gender: Male, age: 23, height: 187 cm, body mass: 70 kg, lifestyle: Standing, physical condition: Average, knee injuries: No, other remarks: Gym (Figure A5);

6. gender: Male, age: 37, height: 189 cm, body mass: 88 kg, lifestyle: Sedentary-standing, physical condition: Average, knee injuries: Rupture of cruciform ligaments in the right knee, other remarks: Cycling, gym (Figure A6);

7. gender: Male, age: 24, height: 183 cm, body mass: 83 kg, lifestyle: Active, physical condition: High, knee injuries: No, other remarks: Swimming and skiing (Figure A7);

8. gender: Female, age 54, height: 164 cm, body mass: 90 kg, lifestyle: Sedentary-standing, physical condition: Average, knee injuries: Right knee injury related to skiing, other remarks: Swimming, irregularly; spinal disc herniation at L2–L3 level (Figure A8);

9. gender: Male, age: 51, height: 184 cm, body mass: 102 kg, lifestyle: Active, physical condition: High, knee injuries: Right knee arthroscopy, ACL and medial meniscus resection, other remarks: Strength sports and alpine skiing (Figure A9);

10. gender: Female, age: 83, height: 158 cm, body mass: 62 kg, lifestyle: Active, physical condition: Average, knee injuries: No, other remarks: Gymnastics and aqua-aerobics (Figure A10);

11. gender: Female, age: 55, height: 160 cm, body mass: 59 kg, lifestyle: Active, physical condition: High, knee injuries: No, other remarks: Gymnastics and cycling (Figure A11);

12. gender: Female, age: 25, height: 164 cm, body mass: 54 kg, lifestyle: Active, physical condition: High, knee injuries: Knee injury (unspecified), other remarks: Running and strength sports (Figure A12);

13. gender: Female, age: 23, height: 162 cm, body mass: 67 kg, lifestyle: Sedentary-standing, physical condition: Average, knee injuries: No, other remarks: Gym since six months (Figure A13);

14. gender: Male, age: 42, height: 189 cm, body mass: 100 kg, lifestyle: Active, physical condition: Average, knee injuries: Numerous injuries and surgeries of the knee joints, other remarks: Cycling and motorbike riding (Figure A14).
